# A modeller's guide for biomedical discovery

**DOI:** 10.1242/dmm.052807

**Published:** 2026-06-23

**Authors:** Philip Greulich

**Affiliations:** ^1^ School of Mathematical Sciences, University of Southampton, Southampton SO17 1BJ, UK; ^2^ Institute for Life Sciences, University of Southampton, SO17 1BJ, UK

## Abstract

Mathematical and computational modelling can do far more than reproduce experimental data or make predictions. When used with intent, models become instruments of discovery: they translate qualitative biological ideas into quantitative, testable hypotheses; they connect microscopic questions to macroscopic data; and, crucially, they help falsify plausible but incorrect mechanistic narratives. This Perspective explores how modelling achieves these goals. I begin by outlining why classical experimental strategies and conventional statistics sometimes fall short of addressing the mechanisms we care about. I then present a model-centred workflow for scientific discovery, using clonal lineage tracing as a running example. The second half focuses on a phenomenon that both limits and empowers model inference – universality – and explains how to turn it from a curse into an opportunity. I conclude with a concise, practical guide that distils these ideas into steps for day-to-day biomedical research.

## Teaser

**Summary:** Mathematical modelling is not simply data fitting. Done well, it is a tool for hypothesis testing that proceeds by successively excluding incorrect models rather than by claiming one well-fitting model to be true.

## Introduction

Quantitative models have guided biological research since the early 20th century, from epidemiology (Kermack and McKendrick, 1927) to population genetics (Kolmogorov et al., 1937) and the randomness of mutations (Luria and Delbrück, 1943), and they have since enriched developmental and stem cell biology (Turing, 1952; Mogilner et al., 2006; Tomlin and Axelrod, 2007; Chaplain, 2011; MacArthur, 2023). A landmark example of how mathematical models can drive biological research is the finding by Luria and Delbrück that mutations arise randomly, rather than in response to selection pressure (Luria and Delbrück, 1943). This finding could not have been made without a mathematical model, because the experimental data available at the time could not directly address the biological question and did not allow the competing hypotheses to be tested directly. Instead, Luria and Delbrück compared predictions from different versions of the model and showed that only the version in which mutations occur randomly, without selective pressure, is consistent with the data. Crucially, they did not attempt to reproduce every biological and biochemical detail of mutation and selection. Instead, they isolated the basic population-level processes needed to formulate the hypotheses mathematically and to make robust quantitative predictions.

Mechanistic quantitative modelling is useful not merely to reproduce data, but to connect biological mechanisms to available measurements by generating virtual data from the hypotheses behind those mechanisms that can be directly compared with experiments, even when the hypotheses themselves cannot. Agreement between real and modelled data, however, does not prove the hypothesised mechanism correct. Indeed, a key lesson of this Perspective is that such agreement often does not single out a unique mechanism; rather, it is disagreement that allows us to reject alternative possibilities and narrow the hypothesis space. In this sense, modelling is fundamentally about ruling things out.

## Why experiments and statistics alone are sometimes not enough

Three recurring gaps often separate the biological questions we ask from the datasets we can feasibly collect:

- Temporal gap. Many questions concern dynamic processes, such as how cells divide and differentiate over time; yet, *in vivo* measurements are often limited to static snapshots (such as immunostaining of fixed tissue samples or single-time-point gene-expression measurements) (Kretzschmar and Watt, 2012; Weinreb et al., 2018). Data are captured at discrete time points, often from samples of different origins, requiring an explicit dynamical framework to integrate information across time and cell populations. Although intravital live imaging has made substantial progress in recent years, many tissues and biological questions remain outside its scope because of imaging complications, ethical constraints and the limitations of live fluorescent markers (Coste et al., 2020; Hor and Germain, 2022; Scheele et al., 2022).
- Scale gap. We are often interested in single-cell behaviour, whereas readouts typically reflect cell populations at the tissue level.
- Transformation gap. Hypotheses are expressed as rules, not directly in terms of observables, and the mapping between them is rarely obvious.

A model addresses all three by acting as a translator. It accepts hypotheses formulated as rules, evolves them forward – as computer simulations or by solving mathematical equations – and outputs predicted observables in the same format as our measurements. In this way, models can convert hypotheses that cannot be directly assessed experimentally into measurable predictions.

## How modelling enables hypothesis testing

The discovery workflow begins with hypotheses framed as rules. These rules can be encoded mathematically as systems of differential equations or as random (stochastic) processes. The model is then simulated or analysed to obtain predicted distributions of observables under the assumption that the hypothesis is true.

The central test is the comparison of predictions with real data through parameter reduction [e.g. rescaling or non-dimensionalisation (Schaeffer and Cain, 2016; Castro and de Boer, 2020)] and model fitting. If no parameter choice allows predictions to match the data within statistical uncertainty, the hypothesis is rejected. If a match is possible, the hypothesis remains viable. Nonetheless, more than one hypothesis may generate predictions that are consistent with the data. Consistency with the data, therefore, does not confirm a hypothesis; it shows only that the hypothesis remains a viable candidate. Discovery, then, proceeds by progressive exclusion: we test multiple competing hypotheses, each translated into its own model, and identify the minimal set of hypotheses consistent with the data. This follows the classical scientific method as articulated by Popper (1959).

Modelling is powerful, but it has clear limits. The first challenge is to identify plausible candidate models. This relies on prior biological knowledge and scientific intuition and is, at least initially, an informed and disciplined guess. The challenge becomes substantially harder when many real-world features are incorporated in an attempt to make a model more ‘realistic’, as this typically leads to highly complex structures and an overwhelmingly large space of possible models.

A second, closely related challenge is overfitting (Lever et al., 2016): when models contain many free parameters that must be inferred from limited data, multiple parameter combinations – or even incorrect model structures – may fit the data equally well. This can result in misleading parameter estimates and erroneous conclusions about the underlying mechanism. A third challenge is non-identifiability (Wieland et al., 2021): even relatively simple models may not be uniquely determined by the data. Structural non-identifiability occurs when different models make identical predictions by construction (Wieland et al., 2021; Browning et al., 2024), while practical non-identifiability arises when predictions differ in principle but cannot be distinguished given experimental noise, or when predictions become too similar in macroscopic regimes or at large times (Wieland et al., 2021). Consequently, even models that fit the data well may be mechanistically wrong.

Two principles, therefore, keep this modelling approach stringent. First, parsimony: include only the parameters and features needed to connect hypothesis to data, because unnecessary model complexity increases the risk of overfitting, such that models may fit idiosyncrasies of the training data rather than the underlying signal (Lever et al., 2016). Second, follow the Popperian scientific method (Popper, 1959): design experiments and tests with the goal of falsification, not confirmation. In this way, we avoid the fallacy of accepting well-fitting models that are nonetheless wrong, even if another model – possibly not yet tested – fits the data equally well while better reflecting the underlying mechanism.

A clear illustration of a Popperian modelling strategy is provided by several recent studies that explicitly formulated and rejected competing mechanistic hypotheses. Johnston et al. (2015) compared competing mechanisms for the mitochondrial DNA bottleneck – a process central to the inheritance of mitochondrial disease – and found strongest support for a birth–death–partition mechanism over alternative models. Cholewa-Waclaw et al. (2019) applied a similar approach to neuronal gene regulation in the context of Rett syndrome, a neuronal genetic disease. They formulated alternative models in which DNA methylation and MECP2 affect transcription either through switching genes on and off, limiting transcription initiation or RNA polymerase II elongation dynamics. Stochastic traffic-based models were used to generate predictions for polymerase occupancy and transcriptional output, which were compared with genome-wide RNA-sequencing and chromatin immunoprecipitation sequencing (ChIP-seq) data, excluding gene-toggle and initiation-based mechanisms.

## Case study: clonal cell lineage tracing

In stem cell, cancer and developmental biology, a recurring question is how stem cells balance self-renewal with differentiation (cell-fate choice) (Clayton et al., 2007; Lopez-Garcia et al., 2010; Simons and Clevers, 2011; Doupé et al., 2012; Blanpain and Simons, 2013; Driskill and Pan, 2023). The question is at what rate or with what probability stem cells divide, and what fate the daughter cells adopt: do they both retain the identity of the mother cell (symmetric replication), do they both differentiate into one or more possible differentiated cell identities (symmetric differentiation), or does one retain the mother's identity while the other does not (asymmetric division)? *In vitro*, these events can be directly observed, but in their native *in vivo* environment – which is essential for reproducing genuine cell-fate dynamics – this is often difficult. A canonical *in vivo* approach (which can also be applied to *in vitro* systems) is clonal cell lineage tracing. First, cells in a tissue are labelled sporadically in space [common methods include genetic labelling through recombination (Sauer, 1998; Soriano, 1999), viral barcoding (Kebschull and Zador, 2018; Bramlett et al., 2020) and CRISPR-based techniques (McKenna et al., 2016; Xie et al., 2023)]. Their progeny then inherit the label, forming ‘clones’, the descendants of the originally labelled single cells. At chosen time points, tissues are harvested and scored: the number of labelled cells per clone and their differentiation status (if measurable) are recorded (Kretzschmar and Watt, 2012), and the clonal frequencies are mapped as clone-size distributions. The dataset is rich but indirect: we observe population-level clone outcomes, not the single-cell fate decisions that produced them, and only as static snapshots.

Now, three hypotheses can be considered (Greulich and Simons, 2016):

- Hypothesis A. Cell fate is variable yet decided at cell division: stem cells may divide symmetrically (both daughter cells retain the naïve stem-cell state or both differentiate) or asymmetrically (one daughter cell retains the naïve stem-cell fate and the other differentiates) and fate is irreversible; that is, a return to the naïve stem-cell state is not possible upon differentiation.
- Hypothesis B. Divisions are strictly asymmetric, but fate is reversible: one daughter cell retains the naïve stem-cell state and cannot differentiate further, while the other daughter cell either proceeds to differentiation or reverts to the naïve stem-cell state (reversible fate choice).
- Hypothesis C. Invariant asymmetric divisions: each division yields one daughter cell in the naïve stem-cell state and another daughter cell that irreversibly differentiates.

**Fig. 1.**
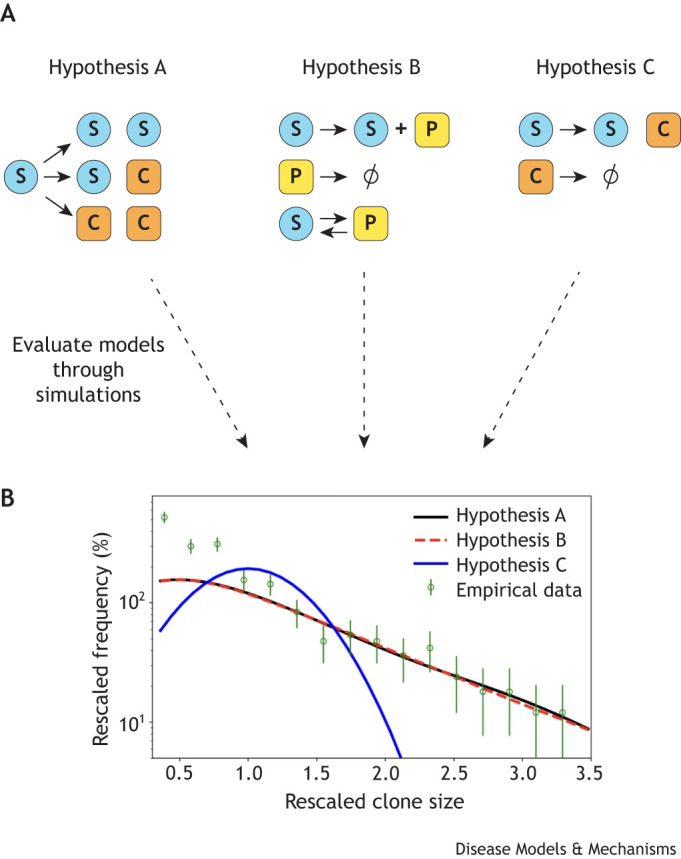
**Modelling cell-fate choices.** (A) Three hypotheses [rules of (stochastic) model] describing possible cell-fate choices. Here, ‘S’ denotes (naïve) stem cells, ‘P’ denotes progenitor cells with the potential to revert to the naïve stem-cell state, and ‘C’ denotes committed cells, which cannot revert to the naïve stem-cell state. The arrows denote changes in cell configuration, and ‘∅︀’ denotes the absence of a cell. Each hypothesis is encoded as a set of stochastic transition rules and implemented as a Markov model. (B) Testing three hypotheses by simulation. Starting from a single cell, each model simulates virtual clones under each hypothesis and scores their sizes to obtain predicted clone-size distributions. The graph overlays the resulting rescaled clone-size distributions – plotted as probability/frequency density (normalised such that the area under the curve equals one) of the rescaled clone size (clone size divided by the mean clone size) – with the empirical data from lineage tracing in mouse oesophagial epithelium by Doupé et al. (2012). Hypotheses A and B yield indistinguishable predictions that agree with the data, whereas Hypothesis C does not.

These hypotheses are depicted schematically as sets of rules in Fig. 1A. The problem is that they cannot be tested directly against the data: the hypotheses concern the rules of a single cell division, or single-cell state changes, whereas the data comprise multi-cell clones observed only as static snapshots. Yet from the sets of rules stated in the hypotheses, one can construct stochastic models. These models can be evaluated mathematically or via simulation, starting with a single cell and applying the rules repetitively to predict populations, that is, clones. From this, we generate predicted virtual clone-size distributions (Fig. 1B).

When the predictions are compared with measured data, something striking occurs: predictions from both Hypotheses A and B fit the data. Only Hypothesis C produces a distinct prediction, failing to reproduce the data and therefore being excluded. This is surprising at first glance. Looking only at the biological features, one might expect Hypotheses B and C to be more similar, because both involve asymmetric divisions only, whereas only Hypothesis A includes symmetric divisions.

**Fig. 2.**
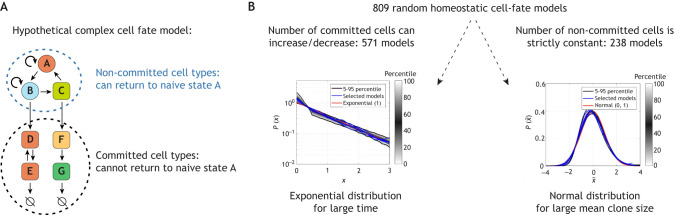
**Universality of clone-size distributions from random homeostatic cell-fate models.** Reproduced from Parigini and Greulich (2020) under the terms of the CC-BY 4.0 licence. Simulation of 809 models comprising cell types that may or may not divide, (trans-)differentiate or be lost (that is, die, be shed or emigrate), and cell types classified as non-committed (non-zero probability of reverting to the naïve state) or committed (cannot revert to the naïve state; progeny are inevitably committed to loss). (A) An example hypothetical cell-fate model with complexity greater than that of the models introduced in Fig. 1. Letters represent different hypothetical cell types; arrows indicate possible transitions [e.g. by (de-)differentiation] between cell types, either directly or via cell division. ‘∅︀’ denotes the absence of a cell. Non-committed cell types are shown in the blue dashed line circle; committed cell types are in the black dashed line circle. (B) 809 randomly generated homeostatic cell-fate models (with cell-type transitions and divisions randomly selected for each model) were simulated, and their clone-size distributions were recorded and plotted against the rescaled clone size *x*=*n*/<*n*>, and against the *z*-score *x̃*=(*n*−<*n*>)/σ, where *n* is the clone size, <*n*> is the mean clone size, and σ is the standard deviation. When the number of non-committed cells is not conserved (571 models), the clone-size distribution converges to an exponential distribution (left). When the number of non-committed cells is strictly conserved (238 models), the distribution is peaked and, for large mean clone size, approaches a normal distribution (right). Small clone sizes also lead to peaked distributions, but these are not strictly normal (Parigini and Greulich, 2020). Shaded bands indicate fifth to 95th percentiles across models; solid line curves show representative examples.

To understand this unexpected result, let us consider a previous study of modelling cell-fate choice (Parigini and Greulich, 2020). In that study, hundreds of stochastic models of cell division and fate choice, under the condition that they be in homeostasis, were simulated (Fig. 2). These models included more parameters than the example hypotheses above, including multiple sets of cell types or states, division events and fate transitions between them (more than the two in the hypotheses above; see, for example, Fig. 2A). Notably, many models produced the same exponential clone size distribution when matched by mean clone size and, thus, matched the data equally well (Fig. 2B). Other models produced distributions that, for large mean clone sizes, converged to a normal distribution (Fig. 2B). This allowed us to distinguish two model classes: Class 1, models with exponential clone-size distributions when dynamics run for a long time; and Class 2, models with normal distributions at large mean clone sizes.

The predictions of models within the same class were indistinguishable. The important question is: what united this plethora of seemingly different models? To answer this, let us classify cells into two categories: non-committed cell types, which can – with some probability – revert to the original naïve state; and committed cell types, which cannot revert to the original naïve state and the progeny of which will eventually be lost (by death, shedding or migration out of the tissue of interest). If we classify the models described above according to whether the number of non-committed cells is strictly conserved and then sort them into either Class 1 or Class 2, we find that models in which the number of non-committed cells is not strictly conserved fall into Class 1, and models in which the number of non-committed cells is strictly conserved fall into Class 2.

Notably, Hypotheses A and B both fall into Class 1. In Hypothesis A, cells labelled ‘S’, but not cells labelled ‘C’ (Fig. 1A), are non-committed, and their number is not conserved because both daughter cells may retain the naïve stem-cell state, allowing the number of non-committed cells to increase upon division. In Hypothesis B, both cells labelled ‘S’ and ‘P’ (Fig. 1A) are non-committed, because P cells can revert to the naïve stem-cell state. Thus, despite the apparent asymmetry, the number of non-committed cells in Hypothesis B is also not conserved, because it can increase through a division event that produces an S cell and a P cell. Hypothesis C, by contrast, strictly conserves the number of S cells – the only non-committed cell type in that hypothesis – and therefore falls into Class 2, where it fails to fit the data.

Despite their different rules, Hypotheses A and B share the absence of a conservation law, because in both hypotheses the number of non-committed cells is not conserved, and both therefore belong to Class 1. Hypothesis C, by contrast, does have a conservation law – the number of non-committed cells is conserved – and therefore belongs to Class 2. This phenomenon, in which distinct mechanisms yield indistinguishable predictions because they share a structural feature, is a form of model non-identifiability and is, in this context, called universality.

## Universality: what it is and why it matters

Universality means that distinct microscopic mechanisms can generate the same macroscopic observables once the system is viewed on the appropriate scale – for example, after rescaling by the mean – and at sufficiently long times, or in sufficiently large systems (Kadanoff, 2000; Rulands and Simons, 2017; Rulands et al., 2018). In practical modelling terms, universality is therefore a form of weak identifiability: models remain mechanistically different; yet, under the conditions and measurements available in practice, their predictions collapse onto the same limiting prediction (Wieland et al., 2021).

There are at least three closely related ways in which such universality arises.

- Asymptotic weak convergence of stochastic processes (Billingsley, 1999): when an observable is the aggregate outcome of many small (stochastic) events, its distribution can converge to a limiting form that forgets much of the microscopic detail. The normal distribution is the canonical example, and this is one reason why normal approximations are so widespread in statistics (Billingsley, 1999).
- Asymptotic model sloppiness (Gutenkunst et al., 2007): many models based on differential equations have solutions that do not change substantially when certain parameters or model features are varied, and this becomes more pronounced asymptotically for large systems, long times or other extreme parameter values. Such parameters are termed ‘sloppy’, and the corresponding features are irrelevant for prediction.
- Coarse graining (Kadanoff, 2000; Ódor, 2004): when microscopic degrees of freedom (e.g. individual agents, cells, molecules or particles) are progressively integrated out, many different microscopic models converge to the same effective large-scale description (a process called renormalisation group). This is also reflected close to criticality: near critical points of a system, in which qualitative macroscopic features change (e.g. liquid versus solid), only certain robust features such as dimension, topology and conserved quantities matter for distinguishing models.

Historically, the term universality was used in physics mainly for the latter mechanism, in which very different microscopic systems share the same large-scale theory. In biological settings, the same conceptual toolkit has proven useful even when the underlying mathematics is closer to weak convergence than to textbook critical phenomena. The practical lesson is the same in both settings: universality reveals which model features remain relevant at the observational scale and which are washed out.

An influential biological formulation of this idea was given by Klein and Simons (2011). They showed that although cell-fate rules may differ across tissues, once clone statistics are viewed at the appropriate scale they are governed mainly by coarse features, such as whether stem cells compete for space and the effective dimension of the tissue (e.g. mouse gut being effectively one dimensional, while basal epidermis is effectively two dimensional), whereas much of the tissue-specific molecular detail disappears from the readout.

Rulands and Simons (2017) made this conceptual point explicit and placed it in the broader context. They argued that quantitative lineage tracing does not, and need not, reconstruct every molecular regulatory programme. Instead, by identifying effective variables, scaling forms and universality classes, it provides a rigorous framework for classifying stem-cell self-renewal strategies while remaining agnostic about many underlying molecular details. A particularly striking example comes from Rulands et al. (2018), who studied clonal dynamics during tissue development, when clonal clusters initially fragmented and merged. They showed that this very complication produces its own universal behaviour: for large times, clone-size distributions followed the same characteristic log-normal distribution, independently of the actual cell-fate choices. These works are in line with the above-mentioned study of a vast number of complex cell-fate models (Parigini and Greulich, 2020), which showed that only the question of whether non-committed cells are strictly conserved or not distinguishes long-term predictions for clone-size distributions.

Universality explains why good fits do not imply unique mechanisms. Many models may fit the data, but some of them may not represent the underlying biological mechanism. For example, Hypotheses A and B above both produce exponential clone-size distributions because they share a key feature: a non-conserved number of non-committed cells. Yet, biologically they differ substantially: one corresponds to a one-way differentiation pathway, whereas the other allows reversible transitions, often referred to as ‘dedifferentiation’, ‘priming’ or ‘licensing’ – features that, ideally, we would like to resolve in order to pinpoint stem cells' lineage potential.

## Turning the curse into an opportunity

Universality may seem like a barrier to mechanistic discovery, but it is also a compass. It reveals which features matter for modelling and which do not. If many models share predictive features and lie in the same universality class, we can replace them with a simple representative of that class without losing predictive power. This legitimises simplicity and guides model reduction.

Moreover, universality is one of the reasons modelling works at all. If every detail were relevant for prediction, we would need highly complex models that mirror reality in full, leading to overfitting and rendering hypothesis testing ineffective. Tiny, biologically irrelevant omitted details could cause otherwise correct hypotheses to be rejected. Thanks to universality, we can use models that are sufficiently simple to analyse.

Commonly, when modelling systems with randomness, Markov chains are used. Markov models assume that consecutive events occur at random time points, with rates independent of what happened previously and when it happened (the process ‘has no memory’), which implies exponentially distributed division times. In reality, however, event timings are not generally exponential but could follow all kinds of distributions. For example, cell divisions follow peaked distributions of cell cycle lengths (Pham et al., 2018). Yet universality, specifically weak convergence, often assures that, over time, the distribution of individual events does not matter when variables are appropriately rescaled and frequencies are normalised (Sagitov, 1986; Jagers and Nerman, 1996). This allows us to model real-world processes as Markov processes and opens the door to efficient simulation techniques such as Monte Carlo methods and to established mathematical frameworks for analysing Markov processes. By contrast, non-Markovian processes are generally more difficult to analyse mathematically, because memory effects destroy the simplifying structure and general theory available in the Markov case (Takacs, 1966; Ciobanu, 2023).

Models belonging to different universality classes typically differ substantially in their predictions, often exhibiting distinct quantitative and qualitative features. For example, in the cell fate models shown in Fig. 2, one universality class yields an exponential (monotonically decreasing) distribution, whereas the other produces a peaked distribution. Similar qualitative distinctions have been demonstrated in other universality settings, such as models of merging and fragmenting clones (Rulands et al., 2018), or scenarios in which clonal fate follows cell replacement (Klein and Simons, 2011), as well as in numerous examples from physics. Consequently, the rejection of an inappropriate universality class is often straightforward and does not depend critically on the specific – potentially ambiguous – choice of goodness-of-fit metrics.

But how do we distinguish between models in the same universality class? Universality emerges only under specific conditions: long times, large system sizes or extreme parameter values. In practice, three levers help break universality:

- Change when you look. Universal behaviours often emerge only at long times. One can therefore shorten experiments, for example, by taking lineage-tracing measurements earlier, to measure clone-size distributions before convergence has occurred (Alcolea et al., 2014; Frede et al., 2016).
- Change what you measure. Adding modalities: spatial context, cell-state markers or lineage barcodes can expose features that were previously invisible. For example, to distinguish Hypotheses A and B, above, explicit immunofluorescent markers and cell-type-specific clonal induction of P-type cells could make reversible fate transitions from P to S directly observable.
- Get information from elsewhere. Some parameters, such as the cell-division rate, may be measured using other experimental approaches, for example H2B-GFP dilution assays (Foudi et al., 2009; Frede et al., 2016).

In practice, deciding how best to deploy these strategies requires additional care. For example, changing the observation times from long to short – and therefore performing measurements before convergence to universality has occurred – also means losing some of the useful consequences of universality: the Markov approximation (in which stochastic event times occur independently) becomes less accurate, because other details of the models become too relevant, which undermines the simplicity of the model and increases the risk of overfitting. Therefore, deciding on the right experimental regimes often requires trade-offs between model identifiability and complexity.

To address the challenges posed by universality and to optimise the data and modelling campaign, we require some initial ‘data-free’ theoretical model analysis before experiments begin. By analysing candidate models in regimes in which universality is suspected, we can identify the few features that define classes and the measurements that would distinguish them.

## Data-free model analysis: the engine room of discovery

Models are often seen as tools for fitting data. Yet, in the discovery workflow, their greatest value lies in preliminary analysis. Even without data, we can do the following:

- Identify universality classes, and find which features are predictively relevant and predictively irrelevant for a universality class: predictively relevant features are those that lead to different predictions when changed, and thus distinguish universality classes, whereas predictively irrelevant features are those that do not affect predictions in the universality classes limiting conditions. There is no unique one-size-fits-all method for doing this, but useful approaches include the renormalisation group (Kadanoff, 2000) (for classical universality in many-agent systems), Markov approximations of branching processes (as in Parigini and Greulich, 2020) and asymptotic theory (Olver, 1997; Le Cam and Yang, 2000).
- Identify internal inconsistencies in candidate models, which allow models to be excluded before testing. For example, Greulich et al. (2021) showed that models lacking a non-committed cell type at the lineage apex cannot be homeostatic.
- Uncover logical relationships between hypotheses, in which hypotheses may mutually imply or exclude each other. For example, in Greulich et al. (2021), it was also demonstrated that self-renewal and lineage potential imply each other in homeostasis.

This analysis sets the experimental strategy: it tells us which hypotheses to test, which measurements to collect and which parameters to estimate.

For example, in Parigini and Greulich (2020), an initial set of 10,000 random models could immediately, without testing, be reduced to around 800 models, from the knowledge that non-committed cells are always present at the apex of a lineage hierarchy (and only there), a fact established by prior modelling work performed without experimental data (Greulich et al., 2021). Then, using a Markovian approximation, the two universality classes could be determined, reducing more than 800 models to only two classes.

## A guide to discovery

Discovery is an iterative conversation between ideas, models and measurements. The sequence could unfold as follows:

(1) Formulate a comprehensive set of hypotheses, making sure not to miss a candidate that could be true.
(2) Formulate mechanistic mathematical models representing each hypothesis, for example as differential equations, stochastic processes or network models.
(3) Analyse whether any of the models imply one another, are mutually exclusive, or can be excluded *a priori* because of intrinsic inconsistencies or inconsistencies with the circumstances (e.g. a model for homeostasis should not predict increasing cell numbers). In this way, reduce the number of models and hypotheses.
(4) Analyse the models for their universality classes. Do some model classes lead to the same predictions in realistic limiting cases? If so, they can be combined into a universality class (UC), which may possibly include an even wider range of models. Methods for doing this include the renormalisation group, Markov approximations, non-dimensionalisation, asymptotics and brute-force simulation.
(5) Following the identification of UCs, identify predictively relevant features, that is, those that distinguish different UCs and lead to different predictions, and irrelevant features, which lead to the same asymptotic predictions within a given universality class.
(6) Find, for each UC, a minimal model that represents that UC while neglecting all irrelevant features.
(7) Evaluate the minimal models for each UC. Fit them to the data and compare their predictions. Reject models that do not match the data and retain those that do for further analysis.
(8) If only one model is retained, we accept the corresponding UC and retain all hypotheses associated with it. If more than one model – that is, more than one UC – is retained, this means that the data lacked sufficient statistical power (e.g. because of small sample size). Revise the experiments to improve statistical power.
(9) When a UC is accepted, we can revise the hypotheses to see whether a coarser-level hypothesis, combining all hypotheses within that UC, can be formulated and thus accepted. If details beyond those distinguishing UCs are required, we can revise the experiments and ask whether other types of data or different experimental conditions can lead to discernible predictions within a universality class.

**Fig. 3.**
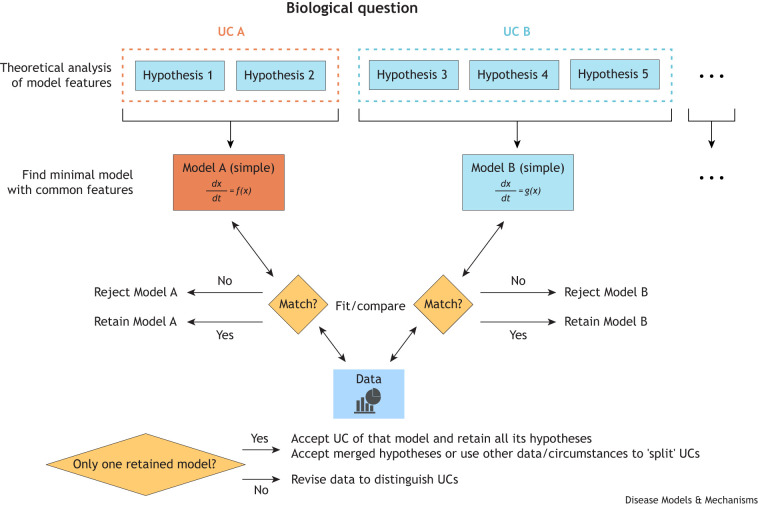
**Illustration of the workflow for using mathematical modelling for biological discovery.** Candidate hypotheses 1,2,3,… are translated into mechanistic models and analysed theoretically to identify universality classes (UCs; A,B,…). For each UC, a minimal representative model is fitted to data; models that do not match the data are rejected, whereas retained models define the supported UC and its associated hypotheses. ‘…’ denotes additional hypotheses or models omitted for visual simplicity. The equations under 'Model A' and 'Model B' are illustrative only. Full description is in the ‘A guide to discovery’ section.

This workflow is illustrated in Fig. 3.

Notably, this full workflow – spanning rigorous theoretical analysis of candidate models, including universality and weak identifiability, through to systematic model rejection in the Popperian sense – has rarely been undertaken in the biomedical modelling field, as the author is not aware of any instance. Looking forward, adherence to this workflow could substantially enhance the reliability and robustness of scientific discovery through modelling.

## Conclusion

Discovery-oriented modelling does not promise unique microscopic truth; instead, it clarifies which features are decidable under given conditions. This clarity prevents wasted effort, guides investment in new measurements and grounds interpretation at the right level, often the level of universality classes.

Modelling for discovery is a disciplined practice. It begins with formulating explicit rules and minimal models, proceeds through theoretical analysis to design decisive experiments and iterates by rejecting models that cannot match the data. Universality is the guiding concept of this process: it explains why multiple mechanisms can fit the same data, legitimises simplicity and points to the measurements that matter.

For disease systems, where the goal is intervention, this approach delivers what we need most: reliable statements about mechanisms that control observable features, such as disease symptoms, which may reveal possible targets for intervention. The path from observation to mechanism and then intervention becomes shorter – not through complexity, but through asking the simplest questions our data can decisively answer.
